# Factors Determining *Staphylococcus aureus* Susceptibility to Photoantimicrobial Chemotherapy: RsbU Activity, Staphyloxanthin Level, and Membrane Fluidity

**DOI:** 10.3389/fmicb.2016.01141

**Published:** 2016-07-19

**Authors:** Monika Kossakowska-Zwierucho, Rajmund Kaźmierkiewicz, Krzysztof P. Bielawski, Joanna Nakonieczna

**Affiliations:** ^1^Laboratory of Molecular Diagnostics, Department of Biotechnology, Intercollegiate Faculty of Biotechnology, University of Gdansk and Medical University of GdanskGdansk, Poland; ^2^Laboratory of Biomolecular Systems Simulation, Intercollegiate Faculty of Biotechnology, University of Gdansk and Medical University of GdanskGdansk, Poland

**Keywords:** photoinactivation, *Staphylococcus aureus*, carotenoids, oxidative stress, RsbU, staphyloxanthin, membrane fluidity

## Abstract

Photoantimicrobial chemotherapy (PACT) constitutes a particular type of stress condition, in which bacterial cells induce a pleiotropic and as yet unexplored effect. In light of this, the key master regulators are of putative significance to the overall phototoxic outcome. In *Staphylococcus aureus*, the alternative sigma factor σ^B^ controls the expression of genes involved in the response to environmental stress. We show that aberration of any *sigB* operon genes in *S. aureus* USA300 isogenic mutants causes a pronounced sensitization (>5 log_10_ reduction in CFU drop) to PACT with selected photosensitizers, namely protoporphyrin diarginate, zinc phthalocyanine and rose bengal. This effect is partly due to aberration-coupled staphyloxanthin synthesis inhibition. We identified frequent mutations in RsbU, a σ^B^ activator, in PACT-vulnerable clinical isolates of *S. aureus*, resulting in σ^B^ activity impairment. Locations of significant changes in protein structure (IS256 insertion, early STOP codon occurrence, substitutions A230T and A276D) were shown in a theoretical model of *S. aureus* RsbU. As a phenotypic hallmark of PACT-vulnerable *S. aureus* strains, we observed an increased fluidity of bacterial cell membrane, which is a result of staphyloxanthin content and other yet unidentified factors. Our research indicates σ^B^ as a promising target of adjunctive antimicrobial therapy and suggests that enhanced cell membrane fluidity may be an adjuvant strategy in PACT.

## Introduction

Growing multiresistance to commonly used antibacterials has become a great danger. There is an established list of “alarm pathogens,” which are responsible for the majority of worldwide mortality and morbidity events resulting from infectious diseases. The list contains methicillin resistant *Staphylococcus aureus* (MRSA). In contrast to the expanding antibiotic resistance, in *S. aureus* as well as in many other microbial pathogens, the number of new classes of antimicrobial drugs has shown limited change. Now, emphasis has been placed on the development of new techniques to avoid multidrug resistance in microorganisms, which can either be applied alone or used in combination with classical antibiotics (Cassidy et al., 2012). One such alternative for classical antibiotic treatment is photoantimicrobial chemotherapy (PACT). The bacteria studied so far has not developed resistance to PACT treatment. PACT not only inactivates microorganisms but also it degrades their external virulence factors, which are released outside the cell (Bartolomeu et al., 2016). PACT constitutes a particular type of stress condition, in which bacterial cells induce a pleiotropic and poorly understood effect. The two photodynamic reactions occur in the cell, with type I leading to generation of oxygen radicals and subsequent reactive oxygen species and type II resulting in singlet oxygen (^1^O_2_) formation. Both types are intertwined, and the predominance of one depends on oxygen availability or a photosensitizer (PS) (Wainwright, 1998). The most frequently used singlet oxygen-generators include cationic phenothiazinium derivatives (i.e., toluidine blue O); xanthene dyes derived from fluorescein (i.e., rose bengal); and macrocyclic dyes based on tetrapyrrole structure, such as neutral or cationic porphyrins (i.e., protoporphyrin IX and TMPyP), metallo-phthalocyanines or chlorins (Wainwright, 1998; Cieplik et al., 2014). On the other hand, effective oxygen radicals producers, such as ball-shaped fullerenes or a new class of curcumins and imidazoacridinone derivatives, are available for PACT (Taraszkiewicz et al., 2013; Cieplik et al., 2014).

Considering a “perfect photosensitizer” for antimicrobial chemotherapy, a set of criteria exists, which must be matched as closely as possible, including high ^1^O_2_ quantum yield, high binding affinity to microorganisms and low affinity to mammalian cells, low cytotoxicity and mutagenicity and the ability to efficiently absorb near-red light wavelengths (Cieplik et al., 2014). To date, no such PS has been developed, which would be potent toward all human pathogens. As regards *S. aureus* and other drug-resistant pathogens, we have to face a phenomenon of strain-dependent response to PACT of yet unexplored molecular background (Grinholc et al., 2008). On the other hand, shuffling of appropriate photosensitizers can lead to eradication of strains resistant to one type of PS with another potent compound (Kossakowska et al., 2013). However, the lack of knowledge about primary targets of particular PSs hampers the elucidation of a universal pattern of PS action in all *S. aureus* strains. Some of the last developments in the field points proteins as the major targets of photosensitization with tri- and tetra-cationic porphyrins in *Staphylococcus warneri* (Alves et al., 2015) but phospholipids and polysaccharides were also affected (Alves et al., 2016).

Instead of searching for a perfect PS, one can suppose the existence of a “perfect strain” that can be easily killed with the use of virtually any PS. From that opposite perspective, a hypothetical strain would present a particular molecular signature or a subset of accessory features sensitizing it to PACT. To date, two “omics” approaches have been implemented to characterize global changes in bacterial cells upon photodynamic treatment. These include a proteomic analysis of *S. aureus* subjected to irradiation with tetra-cationic porphirine (Dosselli et al., 2012). As a result, proteins engaged in anaerobic metabolism were identified as PACT targets, thus suggesting the selective impairment of catabolic pathways after oxygen consumption, leading to the lack of energy supply upon treatment. A second study was based on lipidomic analysis of *Staphylococcus warneri* treated with tri-cationic porphyrin (Alves et al., 2013). As primary targets of PACT, the identified membrane phospholipids showed overall modifications in the relative amount of phospholipids and the formation of lipid hydroxides and hydroperoxides, resulting in cell death.

Because photooxidation results in pleiotropic changes within a cell, key master regulators are of putative significance to the overall phototoxic outcome. Among bacterial species, a limited number of master regulators, acting mainly as transcription factors, forms a complex connection network with multiple target genes and controls expression of large regulons. In *S. aureus*, the alternative sigma factor σ^B^, acting as a subunit of RNA polymerase, controls expression of over 150 genes involved in the environmental stress response (Gertz et al., 1999; Senn et al., 2005), transmembrane transport, envelope composition, intermediary metabolism, virulence regulation (e.g., *sarA*) and adhesion to host cells and tissues (Müller et al., 2014). A *sigB* operon and its regulation on a protein level have been described to mirror a *Bacillus subtilis* model, in which σ^B^ also regulates a large set of general stress genes, including those involved in protection against oxidative stress, and also a response of glucose-starved cells (Engelmann and Hecker, 1996; Petersohn et al., 2001; Hecker et al., 2007). Comparison of the σ^B^ activation models in *B. subtilis* and *S. aureus* revealed similarities in the primary regulation of σ^B^ by the anti-σ factor RsbW and anti-anti-σ factor RsbV. Under non-stress conditions σ^B^ remains inactive in a complex with RsbW. Upon titration with activated (dephosphorylated) RsbV, RsbW dissociates from RsbW-σ^B^ complex. The released σ^B^ protein acts further as a master regulator of σB-dependent genes. Based on the protein-protein interaction studies it is known that RsbU activates (dephosphorylates) RsbV thus positively regulating σ^B^ activity (Senn et al., 2005). In *B. subtilis* however, RsbU acts interchangeably with a second phosphatase RsbP. Both PP2C-type phosphatases are elements of two independent signaling cascades induced by physical (RsbU) and nutritional stress (RsbP). In addition, the upstream activating module RsbX-RsbS-RsbT provides a partner-switching interaction of a macromolecular complex, the stressosome, which is absent in *S. aureus* (Senn et al., 2005; Hardwick et al., 2007).

In the present research, we investigated the role of the staphylococcal σ^B^ master regulator in response to PACT with the use of six different photosensitizers and observed a pronounced sensitization of *sigB* operon mutants to intracellularly acting PSs of high singlet oxygen yield. Analysis of PACT-vulnerable clinical isolates, including MRSA and MSSA, revealed frequent aberrations within the σ^B^ activator RsbU, resulting in abolished protein activity. To illustrate the location of identified mutations, we created a theoretical model of the *S. aureus* RsbU protein. In strains presenting undisturbed σ^B^ activity, we propose a highly fluid cell membrane as a hallmark of vulnerability to PACT with selected photosensitizers.

## Materials and methods

### Bacterial strains and growth conditions

Clinical *S. aureus* strains were characterized by Gram staining and the ability to produce coagulase and the clumping factor using Slidex Staph Plus (bioMèrieux, France). All isolates and reference strains used in the study are listed and described in Table 1. Bacterial cultures were grown aerobically in a nutrient trypticase soy broth TSB (bioMèrieux, France) at 37°C with shaking (150 rpm). SA147 cultures were supplemented with 100 μg/ml erythromycin.

**Table 1:** **Reference and clinical ***S. aureus*** strains used in the study**.

**Strain**	**Description**	**Reference**
USA300 (JE2)	Wild type CA-MRSA reference strain USA300_FPR3757.	Network of Antimicrobial Resistance in *Staphylococcus aureus* (NARSA) program
*ΔsigB*	Contains inactivated gene of the RNA polymerase sigma factor σ^B^.	
*ΔrsbW*	Contains inactivated gene of the anti-σ^B^ serine-protein kinase RsbW.	
*ΔrsbV*	Contains inactivated gene of the anti-anti-σ^B^ factor RsbV.	
*ΔrsbU*	Contains inactivated gene of the positive σ^B^ regulator protein RsbU.	
SH1000	A pigmented *rsbU*^+^ derivative of the 8325-4 strain.	Horsburgh et al., 2002
RN6390	A non-pigmented derivative of the 8325-4 strain containing a natural 11-bp deletion in the *rsbU* gene.	Wright Valderas et al., 2002
SA144	Wild type clinical strain with an intact *crtOPQMN* operon.	Liu et al., 2005
SA145	A non-pigmented *ΔcrtM*-null mutant derivative of SA144.	
SA147	A highly pigmented *crtMN* plasmid-complemented variant of SA145 (*ΔcrtM*-pDCerm*::crtMN*).	
1764/p	MRSA isolate from ambulatory unit (ear).	Provincial Hospital in Gdansk and Hospital in Koszalin (Poland)
6452	MRSA isolate from pediatric surgery unit (wound).	
6987	MSSA isolate from orthopedic unit (wound).	
473	MSSA isolate from surgery unit (wound).	
469	MSSA isolate from orthopedic unit (nose).	
146	MSSA isolate from orthopedic unit (wound).	
5491	MSSA isolate from orthopedic unit (wound).	

### Chemicals

The photosensitizers (PSs) used in the study were protoporphyrin IX diarginate (PPArg_2_) (delivered by the Institute of Optoelectronics, Military University of Technology, Warsaw, Poland), toluidine blue O (TBO), 5.10.15.20-tetrakis(1-methyl-4-pyridinio)porphyrin tetra(*p*-toluenesulfonate) (TMPyP), zinc phthalocyanine (ZnPC) (Sigma-Aldrich, Munich, Germany) and *N*-methylpyrrolidinium fullerene iodide (fulleropyrrolidine, Full) (delivered by ProChimia, Sopot, Poland), and rose bengal (RB) Stock solutions were prepared as follows: 1 mM PPArg_2_, TBO and TMPyP diluted in a sterile double distilled water and kept at −20°C; 10 μM RB diluted in a sterile double distilled water and kept at −20°C, 0.1 mM Full diluted in a mixture of DMSO and water (1:9 v/v) and kept at 4°C; and 1 μM ZnPC diluted in DMSO and kept at room temperature. 1.6-diphenyl-1.3.5-hexatriane (DPH) was purchased from Sigma-Aldrich (Germany). A 2 mM stock solution of this dye was prepared in tetrahydrofuran, and a 4 μM working solution was prepared by adding 100 μl to 50 ml of 0.05 M Tris-HCl (pH 7.6). Residual tetrahydrofuran was removed by gentle flushing with nitrogen (Bayer et al., 2000). A DPH working solution was kept in the dark at 4°C until use but no longer than 2 weeks.

### Photoinactivation experiments

Bacterial strains were cultured for 20–24 h and then diluted with a fresh broth to the density of 0.4 McFarland units (10^7^ CFU/ml). A total 100 μl of each culture was loaded into a 96-well plate and incubated in the dark at 37°C for 30 min, either with or without the addition of PS. The following concentrations of PSs were used: PPArg_2_, TBO, TMPyP–20 μM; RB–0.1 μM; Full–1 μM; ZnPC–5 nM. Then, samples were irradiated with light doses ranging from 10 to 30 J/cm^2^. For the irradiation procedure, LED lamps (SecureMedia, Poland), designed and produced for the Laboratory of Molecular Diagnostics, emitting incoherent red light (615–635 nm, maximal intensity at λ_max_ 627 nm) or green light (500–575 nm, λ_max_ 520 nm) were used. Technical parameters were set as follows: output power of 23.4 mW/cm^2^ and energy dose of 10–30 J/cm^2^. Aliquots incubated in the dark with and without PS served as dark controls. After irradiation with each 10 J/cm^2^ light dose, 10 μl of the samples were taken to perform ten-fold serial dilutions in PBS, ranging from 10^−1^ to 10^−4^. Ten microliter aliquots of each dilution were plated on TSA plates (bioMèrieux, France). After overnight incubation at 37°C, the colonies formed were counted, and the results were analyzed statistically. Each experiment was performed at least in triplicate.

### Photosensitizer uptake

Bacterial cultures (20–24 h) were suspended in PBS buffer to a density of 0.4 McFarland units. Bacterial suspensions containing 5 × 10^7^ CFU in 800 μl volume were centrifuged at 5000 × g for 5 min, washed and resuspended in 800 μl PBS. Suspensions were incubated with 20 μM PPArg_2_ in the dark at 37°C for 30 min and then centrifuged at the described conditions. Absorbance of supernatants was measured at 408 nm in 96-well plates using an EnVision® Multilabel Plate Reader (Perkin-Elmer, USA), and the amount of residual PPArg_2_ was calculated in reference to a calibration curve. The amount of photosensitizer accumulated by the cells was calculated by subtracting residual PPArg_2_ concentrations from the initial concentration, that is 3.2 nmols per 10^7^ cells.

### DNA isolation, sequencing and alignment

Bacterial DNA was isolated from 1 ml of the 24 h culture a using GeneJET™ Genomic DNA Purification Kit (Thermo Scientific, USA), according to the manufacturer's protocol. The DNA concentrations were measured using a NanoDrop™ ND-1000 (Thermo Scientific, USA). The primers used for gene amplification were as follows: for *rsbU* 5′-atctctgcagTTAATTTACTCTTTTTATAATC-3′ and 5′-atcgaattcaTGGAAGAATTTAAGCAAC-3′; for *rsbV* and *rsbW* in one product 5′-TTAGCTGATTTCGACTCTTTCG-3′ and 5′-atcgaattcATGAATCTTAATATAGAAACAACC-3′; for *sigB* 5′-atcgaattcaTGGCGAAAGAGTCGAAATCAGC-3′ and 5′-atctctgcagCTATTGATGTGCTGCTTCTTG-3′ (Senn et al., 2005). PCR was performed with 1 U of *Taq* polymerase (Sigma-Aldrich, Germany) and 100 ng of template DNA. PCR products were purified using a QIAquick™ PCR Purification Kit (Qiagen, The Netherlands), and DNA sequencing was accomplished from both ends by the Sanger dideoxy-chain termination method according to standard (500–800 nt reads) or extra-long run protocols (1000–1100 nt reads), depending on template length. Sequences were analyzed and aligned to the reference AP009351.1 sequence (*S. aureus* Newman) using BioEdit Sequence Alignment Editor 7.2.0 (Hall, 1999).

### RNA extraction and qPCR

Overnight bacterial cultures were added to fresh broth in a 1:100 ratio and cultured to reach mid-logarithmic phase of growth (~2.5 h). For total bacterial RNA extraction, 500 μl of cell suspension of OD_600_ 0.5 density (10^8^ CFU/ml) was mixed with 1 ml of RNAprotect® Bacteria Reagent (Qiagen, The Netherlands) according to the manufacturer's instructions. Bacterial cells were then lysed in 100 μl of buffer containing 20 mM Tris-HCl (pH 8.0), 2 mM EDTA, 1.2% Triton X-100 and 2 U lysostaphin (A&A Biotechnology, Poland) at 37°C for 30 min. Bacterial suspensions were further homogenized in MagNA Lyser instrument (Roche, France) with acid-washed glass beads (Sigma-Aldrich, Germany) for 3 min at 6000 rpm. RNA extraction was performed with an RNeasy® Mini Kit (Qiagen, The Netherlands) according to the manufacturer's protocol, with additional on-column DNase I treatment. The quality of total RNA was evaluated on a 1.5% agarose gel, and its concentration was measured using a NanoDrop™ ND-1000 (Thermo Scientific, USA). First strand cDNA synthesis was performed using a TranScriba Kit (A&A Biotechnology, Poland) with the addition of random hexamers and 100 ng total RNA. For qPCR, the *asp23*-specific primers 5′-AAAGCAAAACAAGCATACGACAATC-3′ and 5′-AGCGATACCAGCAATTTTTTCAAC-3′ and reference 16SrRNA specific primers 5′-TATGGAGGAACACCAGTGGCGAAG-3′ and 5′-TCATCGTTTACGGCGTGGACTACC-3′ were used (Ster et al., 2005). cDNA quantification was performed in 10 μl volume with SYBR Green I containing SG qPCR Master Mix (EURx, Poland), 0.25 μM of each primer and 1 μl of 1:10 diluted cDNA using a 480 Light Cycler® instrument (Roche, France). The following protocol was applied: 3 min of initial denaturation at 95°C and 45 cycles of denaturation at 95°C for 10 s, primer annealing at 62°C for 30 s and elongation at 72°C for 30 s, with melting curve analysis at a range of 60 to 90°C. Normalized values of *asp23* expression in analyzed strains were calculated relative to SH1000 calibrator, based on threshold numbers. Each experiment was performed at least in triplicate.

### *S. aureus* RsbU structure modeling

We used RsbU amino acid *S. aureus* sequence deposited under the GenBank number: AP009351.1 to predict the homology model of this protein.

The main difficulty in predicting the structure is the lack of a single protein structure that has significant sequence identity with the target sequence in the PDB database (Protein Data Base). Therefore, multiple templates were used to improve the quality of the obtained target homology model. Up to now, no data describing the 3D structure of this protein was available. To uncover as much structural detail as possible, we conducted the following bioinformatic analyses: the search for structural homologs in the SWISS−PROT database [http://web.expasy.org/docs/swiss-prot_guideline.html (accessed 30.04.2015)] using BLAST [http://blast.ncbi.nlm.nih.gov/Blast.cgi (accessed 30.04.2015)]; an analysis of the secondary structure using PSIPRED [http://bioinf.cs.ucl.ac.uk/psipred/ (accessed 30.04.2015)], SSPRO (Cheng et al., 2005a) and DISEMBL (Linding et al., 2003); searching for unstructured protein fragments using DISOPRED (Jones and Cozzetto, 2015), DISEMBL (Linding et al., 2003) and DISPRO (Cheng et al., 2005b); the identification of transmembrane helices using TMHMM [http://www.cbs.dtu.dk/services/TMHMM-2.0/ (accessed 30.04.2015)], TOPRED (von Heijne, 1992), HMMTOP (Tusnády and Simon, 1998) and MEMSAT (Jones, 2007); prediction of regions with a coiled-coil structure using COILS (Lupas et al., 1991). Additionally, we searched for evolutionarily conserved domains using Reversed Position Specific Blast (RPS−BLAST) [http://www.ncbi.nlm.nih.gov/Structure/cdd/wrpsb.cgi (accessed 30.04.2015)]. The final homology model of the target sequence was assembled with MODELER (Eswar et al., 2006) software.

### Carotenoid extraction

The bacterial cultures were grown for 24 h. Bacterial cells at 2 × 10^9^ (OD_600_ 2.0 in 5 ml volume) were harvested at 4000 rpm at 4°C for 10 min and washed twice with double distilled water. After centrifugation, pellets were suspended in 1.5 ml of 99% methanol (POCH, Gliwice, Poland) and agitated for 2 h in the dark until bleached. Samples were centrifuged at 10,000 × g at 4°C for 15 min, and the absorbance of the supernatant was measured at 450 nm using a Novaspec II spectrophotometer (Pharmacia Biotech, USA). Each experiment was performed at least in triplicate.

### Cell membrane fluidity assay

Temperature-dependent membrane fluidity was quantified by measuring the fluorescence polarization anisotropy (*r*) of DPH, according to modified protocols described by Bayer and Voss (Bayer et al., 2000; Voss and Montville, 2014). DPH, a lipophilic fluorescent probe, preferentially localizes to hydrophobic (intrinsic) regions of cell membrane phospholipids and emits polarized light upon excitation. The value of fluorescence anisotropy (*r*) reflects unequal intensities of emitted light along different axes of polarization (vertical and horizontal) and therefore indicates the degree of a fluorophore's free movement that is dependent on cell membrane fluidity. Whole-cell suspensions of each bacterial strain were prepared with a density of 4.5 McFarland units (10^8^ CFU/ml) in TSB medium. Suspensions were pelleted by centrifugation (5000 × g, 15 min) and then resuspended in 500 μl of digestion buffer (20% [w/v] sucrose, 0.05 Tris-HCl [pH 7.6], 0.145 M NaCl). The bacterial cell wall was then digested with 0.8 U of lysostaphin (A&A Biotechnology, Poland) in the presence of 3 U of DNase I (EURx, Poland) for 1 h at 37°C (Bayer et al., 2000). Protoplasts were collected by centrifugation (10,000 rpm, 15 min) and resuspended in 200 μl of fresh digestion buffer. The adequacy of cell wall digestion was confirmed by Gram staining. For DPH labeling, protoplasts suspended in digestion buffer were mixed with DPH solution in a 1:1 ratio to obtain 2 μM final concentration and incubated in the dark at 30°C for 45 min. Spectrofluorimeter FP-8500 (JASCO, USA) coupled with Spectra Manager™ software was used for fluorescence intensity and anisotropy measurements. Analysis was carried out in a labeled cell suspension volume of 300 μl, agitated at 200 rpm, in a temperature gradient ranging from 20 to 40°C (ramping rate 1°C per 1 min). Above this temperature, a disruption of labeled protoplasts was observed using fluorescence microscopy. A blank measurement was recorded using the unlabeled cell suspension of each strain separately, at a single initial point, as no significant changes in background fluorescence intensity of unlabeled protoplasts were observed during the whole measurement. Measurement parameters were set up as follows: a vertically polarized excitation wavelength of 360 nm (bandwidth 5 nm) and an emission wavelength of 426 nm (bandwidth 10 nm) analyzed through a rotating polarizer. Signals were measured for 2 sec at each 2.5°C interval. A G factor of 1.2017 was used. Each experiment was performed at least in triplicate.

### Statistical analysis

The results of photodynamic inactivation, carotenoid extraction, photosensitizer uptake and quantitative PCR are presented as the average of at least three independent experiments. Statistical significance was assessed using Student's *t*-distribution method. Grouping of fluorescence anisotropy data included analysis of correlation matrix within a whole temperature range followed by hierarchic clustering based on Ward agglomeration and Manhattan distance calculation methods using STATISTICA 10 software (StatSoft Inc. 2011, USA). Three biological replicates of each *S. aureus* strain were included separately into the analysis.

## Results

### Photokilling of *S. aureus* with PPArg_2_, ZnPC and RB is effective against σ^B^-impaired strains

To assess the impact of *sigB* operon impairment on PACT outcome, we first tested the survival of an isogenic set of USA300 mutants treated with increasing light doses (10-30 J/cm^2^) and various photosensitizers. The results are presented in Table 2 as means of viability reduction for a discriminating light dose of 20 J/cm^2^. Full spectrum of the applied light doses are presented in Supplementary Figure 1. A clear difference in vulnerability to PACT was observed between wild type USA300 and *sigB* operon mutants in the presence of PPArg_2_, ZnPC and RB. Here, the applied treatment was inefficient toward the wild type USA300 but led to almost complete eradication of each σ^B^-impaired mutant. By eradication, we considered a reduction of more than 5 log_10_ units in the number of CFU; thus, the drop below a detection threshold of a method. In the presence of TBO and TMPyP, applied irradiation was ineffective toward both wild type and isogenic mutants. Fulleropyrrolidine, although more potent under the herein applied conditions, revealed no discriminative activity with regards to wild type and mutant strains. To verify this observation in a different genetic background, we used another set of strains: RN6390 and SH1000. In RN6390, the *rsbU* gene contains a natural 11-nt deletion, and this deletion is restored in strain SH1000 (Horsburgh et al., 2002). In this system, the lack of functional RsbU—a σ^B^ activator—led to a similar PACT outcome, as observed previously. We noticed effective killing of strain RN6390 upon PPArg_2_, ZnPC and RB treatments and poor phototoxic activity toward strain SH1000 (Table 2).

**Table 2:** **Phototoxic effect of chosen photosensitizers on ***S. aureus*** reference strains**.

**Strain**	**Mean reduction of survival**^a^ **(log**_10_ **CFU)** ± **SD**					
	**TBO^b^**	**TMPyP^b^**	**PPArg_2_^b^**	**ZnPC^b^**	**RB^c^**	**Full^c^**
USA300	0.29 ± 0.22	1.59 ± 0.36	1.63 ± 0.90	1.28 ± 0.70	2.04 ± 0.58	2.85 ± 0.38
*ΔsigB*	1.04 ± 0.36	1.07 ± 0.35	**5.88 ± 0.10**	**5.50 ± 1.00**	**5.24 ± 1.02**	2.78 ± 0.55
*ΔrsbW*	0.72 ± 0.09	0.78 ± 0.23	**5.63 ± 0.05**	**5.74 ± 0.59**	**5.62 ± 0.48**	2.46 ± 0.69
*ΔrsbV*	0.59 ± 0.17	0.88 ± 0.16	**5.81 ± 0.07**	**5.29 ± 0.77**	**5.64 ± 0.54**	**3.12 ± 0.53**
*ΔrsbU*	0.18 ± 0.04	1.56 ± 0.32	**5.98 ± 0.02**	**5.56 ± 0.68**	**5.62 ± 0.48**	2.35 ± 0.38
SH1000	N/A	N/A	0.25 ± 0.02	1.03 ± 0.46	1.18 ± 0.54	N/A
RN6390	N/A	N/A	**4.30 ± 0.14**	**4.48 ± 0.56**	**5.39 ± 1.11**	N/A

a *The values were calculated by subtracting log_10_ CFU/ml of treated samples from those of untreated controls (0 J/cm^2^; 0 μM PS). At least three biological replicates were used for the calculation of the mean reduction values. SD–Standard Deviation. Underlined values indicate statically significant reduction relative to light control (20 J/cm^2^; 0 μM PS) (p < 0.05). Bold values indicate a bactericidal effect (>3 log_10_ reduction units)*.

b *Photosensitizer excited with the red light (maximal intensity at λ_max_ 627 nm). Concentrations used: TBO, PPArg_2_ and TMPyP 20 μM; ZnPC 5 nM. Light dose applied was 20 J/cm^2^*.

c *Photosensitizer excited with the green light (maximal intensity at λ_max_ 520 nm). Concentrations used: RB 0.1 μM; Full 1 μM. Light dose applied was 20 J/cm^2^*.

*TBO–Toluidine Blue O; TMPyP—5.10.15.20-tetrakis(1-methyl-4-pyridinio)porphyrin tetra(p-toluenesulfonate); PPArg_2_–protoporphyrin IX diarginate; ZnPC–Zinc Phthalocyanine; RB–rose Bengal; Full–N-methylpyrrolidinium fullerene iodide*.

Based on the presented results, we conclude that the *sigB* operon affects PACT efficacy with the use of PPArg_2_, ZnPC and RB in *S. aureus*.

### PACT-sensitive clinical isolates reveal variations in the *rsbU* gene

To analyze the correlation between PACT outcome and σ^B^ activity, clinical *S. aureus* isolates previously described as susceptible or insensitive to protoporphyrin IX-mediated PACT (Grinholc et al., 2008) were included in the analysis in a context of *sigB* operon integrity First, we characterized phototoxic activity of the three analyzed PSs (PPArg_2_, ZnPC, and RB) toward these clinical isolates of *S. aureus* (Table 3). From the obtained data, it can clearly be seen that each of the tested clinical isolates is sensitive to at least two out of three analyzed PSs (Table 3).

**Table 3:** **Phototoxic effect of PPArg_**2**_, ZnPC and RB on clinical ***S. aureus*** strains**.

**Strain**	**Mean reduction of survival**^a^ **(log**_10_ **CFU) ± SD**		
	**PPArg_2_^b^**	**ZnPC^b^**	**RB^c^**
1764/_p_	**5.53 ± 0.01**	**3.49 ± 0.77**	**5.47 ± 0.67**
6452	2.05 ± 0.72	**3.76 ± 0.45**	**3.60 ± 1.32**
6987	**5.29 ± 0.01**	2.89 ± 0.55	**3.37 ± 0.65**
473	**5.45 ± 0.04**	**4.90 ± 0.18**	**5.42 ± 0.93**
469	**3.53 ± 0.55**	**5.77 ± 0.10**	1.69 ± 0.12
146	**4.46 ± 0.30**	**4.56 ± 1.06**	**4.48 ± 1.30**
5491	0.61 ± 0.20	2.94 ± 0.64	2.89 ± 0.31

a *The values were calculated by subtracting log_10_ CFU/ml of treated samples from those of untreated controls (0 J/cm^2^; 0 μM PS). At least three biological replicates were used for the calculation of the mean reduction values. SD–Standard Deviation. Underlined values indicate statically significant reduction relative to light control (20 J/cm^2^; 0 μM PS) (p < 0.05). Bold values indicate a bactericidal effect (>3 log_10_ reduction units)*.

b *Photosensitizer excited with the red light (maximal intensity at λ_max_ 627 nm). Concentrations used: PPArg_2_ 20 μM; ZnPC 5 nM. Light dose applied was 20 J/cm^2^*.

c *0.1 μM RB excited with the green light (maximal intensity at λ_max_ 520 nm). Light dose applied was 20 J/cm^2^*.

*PPArg_2_–Protoporphyrin IX diarginate; ZnPC–Zinc Phthalocyanine; RB–rose Bengal*.

**Figure 1:**
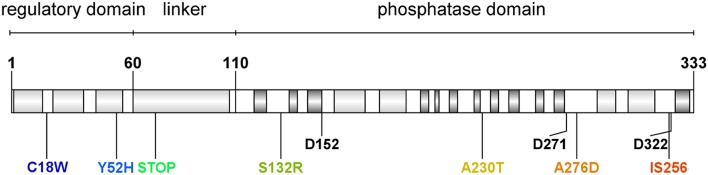
**Domain organization and identified mutations in ***Staphylococcus aureus*** RsbU protein**. Light gray boxes indicate α-helix motifs, and dark gray boxes indicate β-sheet motifs. Aminoacid residues are marked with a single letter amino acid code. Conserved aspartate residues of metal ion binding sites are marked with their position description (D152, D271, D322). Identified aberration sites in analyzed clinical isolates are presented in colors.

We then sequenced the genes of the *sigB* operon (*rsbU, rsbV, rsbW, sigB*) and screened for mutations in these PACT-vulnerable isolates. Interestingly, all the identified mutations were localized to the *rsbU* gene (except for 146 strain, where another N61K substitution in RsbW was also identified). Each PACT-sensitive strain carried different types of variations, which were absent in the less sensitive to PACT strain 5491 (Table 4, Figure 1). The obtained results do not rule out other mutations outside sigB operon as associated with PACT-vulnerable phenotype. Interestingly, the applied irradiation conditions did not lead to PPArg_2_-based eradication of strain 6452 in spite of an obvious aberration within the *rsbU* gene. This phenomenon can be connected to significantly lower PPArg_2_ uptake compared to other analyzed strains (Supplementary Figure 2).

**Table 4:** **The list of aberrations detected in clinical ***S. aureus*** strains in the ***rsbU*** gene**.

**Strain**	**Position in gene^a^**	**Position in protein**
1764/_p_	C827A	A276D
6452	IS256 insertion (1325 nt) in position 960	Insertion in position 321
6987	11 nt deletion in position 218, frameshift and early STOP codon	Deletion from the position of amino acid 76; V74W, K75L
473	G688A	A230T
469	C54G, T396G	C18W, S132R
146	T154C	Y52H

a *Positions of nucleotides are described based on GenBank: AP009351.1 rsbU locus (Staphylococcus aureus strain Newman)*.

### Structural model of *S. aureus* RsbU protein

**Figure 2:**
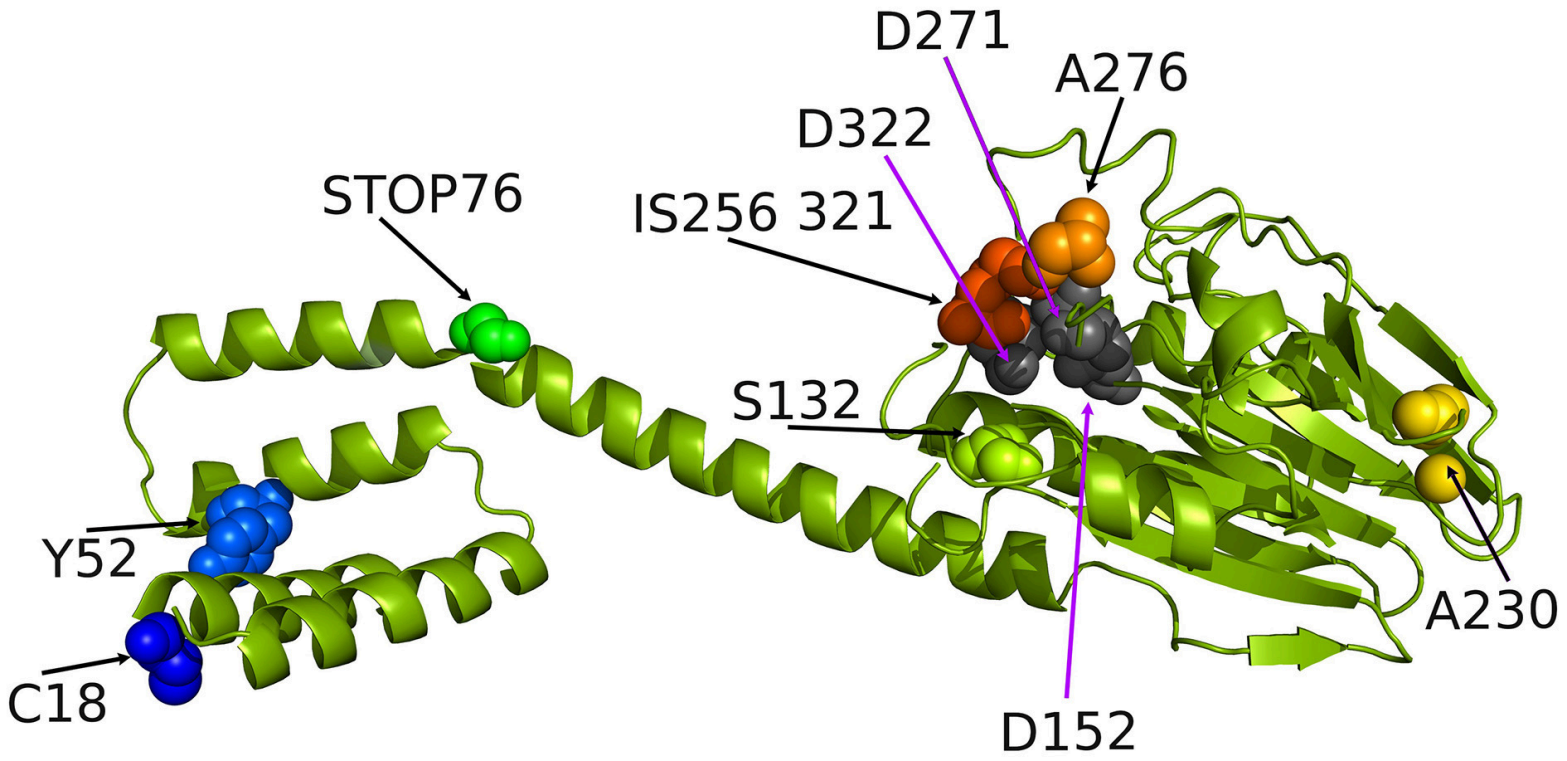
**The ribbon representation of the ***Staphylococcus aureus*** RsbU structure with tagged important residues identified in this study (see the experimental section for explanation)**. C18, Y52, S132, A276, and A230 indicate the position of amino acids identified as mutated in analyzed clinical isolates of *S. aureus*. STOP76 indicates a stop codon in the *rsbU* gene sequence likely resulting in a truncated protein. IS256 321 indicates insertion of an IS256 genetic element in position 321 of the RsbU amino acid sequence.

To date, neither the experimental nor predicted structure of RsbU from *S. aureus* has been published. The crystal structure of the RsbU ortholog in *B. subtilis*, which serves as a model of σ^B^ activation system, has been solved only for the N-terminal regulatory domain (Delumeau et al., 2004). To show the sites of detected mutations within the protein structure and assess their potential relevance, the theoretical structural model of the *S. aureus* RsbU monomer was created and presented with an indication of the identified changes (Figure 2). The results of the bioinformatic analyses allowed us to hypothesize that the 3D structure single-chain conformation of the target sequence consists of the two domains connected with one relatively long α-helix.

Furthermore, the N-terminal domain was found to be all-helical, while the C-terminal domain was predicted to accommodate an αβ-structure. For modeling of the 3D structure of RsbU, we employed two template structures. The first template was the protein structure with the PDB code 3T91 (chain A), in which the 242-residue fragment shared 17% amino acid sequence identity and 42% homologous residues with the target. This template served to predict the RsbU protein fragment from residue 82 to 330. The second template was PDB code 1W53 (chain A), in which the 84-residue fragment shared 29% amino acid residue sequence identity and 54% homologous residues with the target, and it predicted the RsbU protein fragment from residue 2 to 82. We did not predict the conformation of the first residue or the last three residues of the target sequence. The final 3D structure of the RsbU homology model assembled by MODELER (Eswar et al., 2006) consists of two domains: an N-terminal α-helical four helix bundle and C-terminal αββα arrangement, similar to PP2C-type phosphatases (Schweighofer et al., 2004).

The two flanking domains are connected with a long α-helix. Interestingly, both templates form dimers, which may be evidence of a tendency of the target protein structure to form dimers. Dimer formation has also been proposed for *B. subtilis* RsbU, suggesting the localization of dimerization-determining motifs to the N-terminal domain (Delumeau et al., 2004). From the present model, it can clearly be seen that four identified mutations localize in the C-terminal domain, two in the N-terminal domain and one in the linker helix in between the two domains, probably resulting in protein truncation and lack of a C-terminal regulatory domain.

### Some of the identified mutations influence σ^B^ function

**Figure 3:**
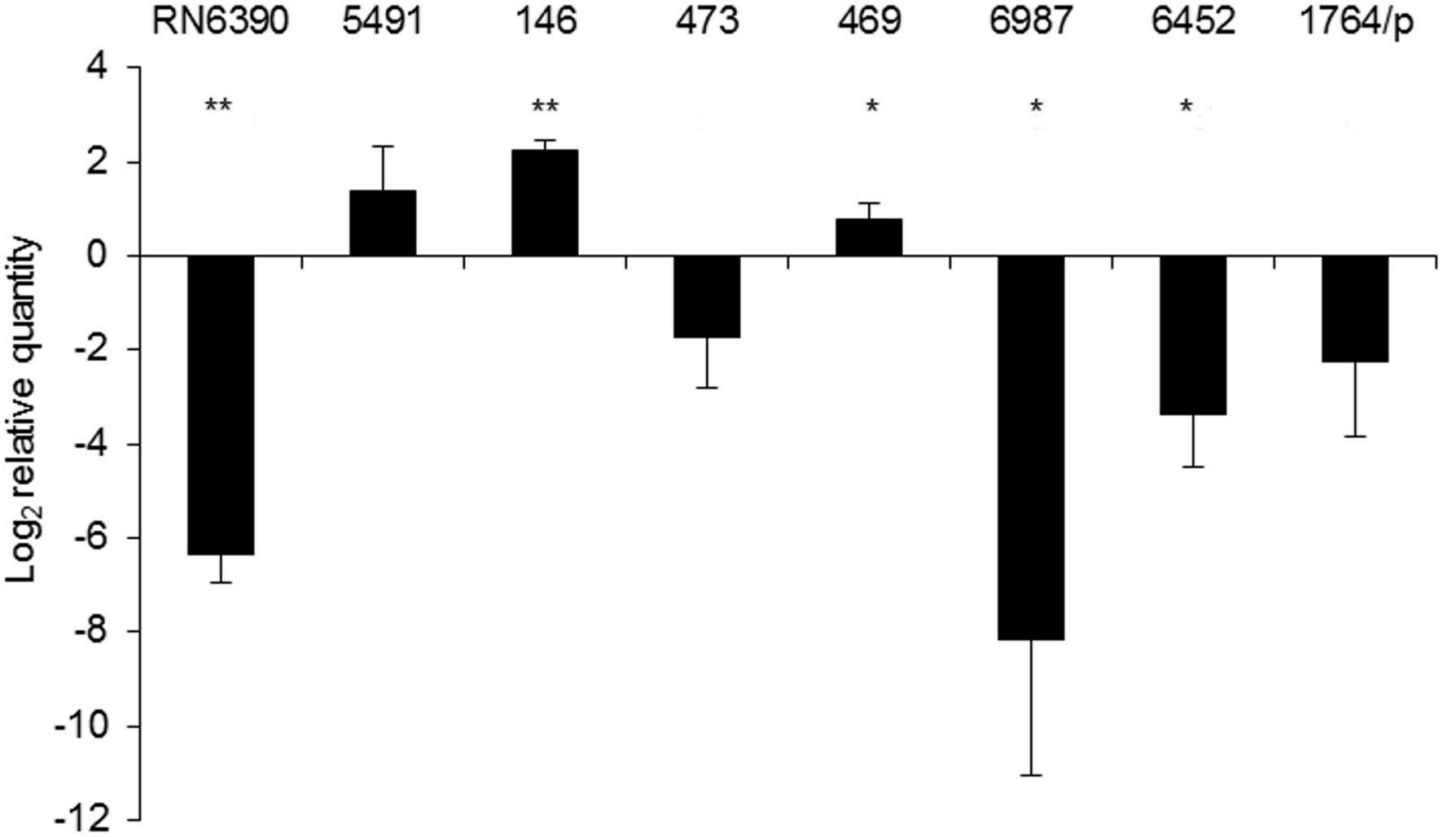
*****asp23*** mRNA levels**. Relative values of *asp23* gene expression were normalized to the 16S rRNA reference gene and calculated relative to SH1000. The value “0” represents the level of asp23 mRNA in the SH1000 strain. The results shown are the average of three independent experiments, and error bars represent standard deviation. Significance at respective *p* values is marked with asterisks (^*^ <0.05, ^**^ <0.01). The names of particular strains are indicated above the bars.

To check if the identified mutations result in a σ^B^-defective phenotype in clinical isolates, a functional test was employed. We analyzed the transcript levels of the membrane-anchored alkaline shock protein Asp23, which is exclusively controlled by σ^B^ factor (Gertz et al., 1999; Müller et al., 2014). The qPCR results were calculated relative to SH1000 transcript levels (Figure 3). Elevated mRNA levels were observed in the clinical isolate strain 5491 with respect to SH1000, in which no missense mutations were detected in the *rsbU* gene. Accordingly, a significant 82.5-fold decrease in *asp23* transcript levels was observed in the RsbU-defective strain RN6390. Four out of six PACT-vulnerable isolates (473, 6987, 6452, 1764/p) showed decreased *asp23* transcript levels, among which 6452 and 6987 revealed statistical significance (Figure 3). On the contrary, elevated transcript levels were observed in strains 469 and 146 compared to SH1000, indicating that the identified substitutions in RsbU (together with N61K substitution in RsbW in strain 146) had no diminishing effect on σ^B^ activity.

### Carotenoid level correlates with σ^B^ activity in all analyzed strains

**Figure 4:**
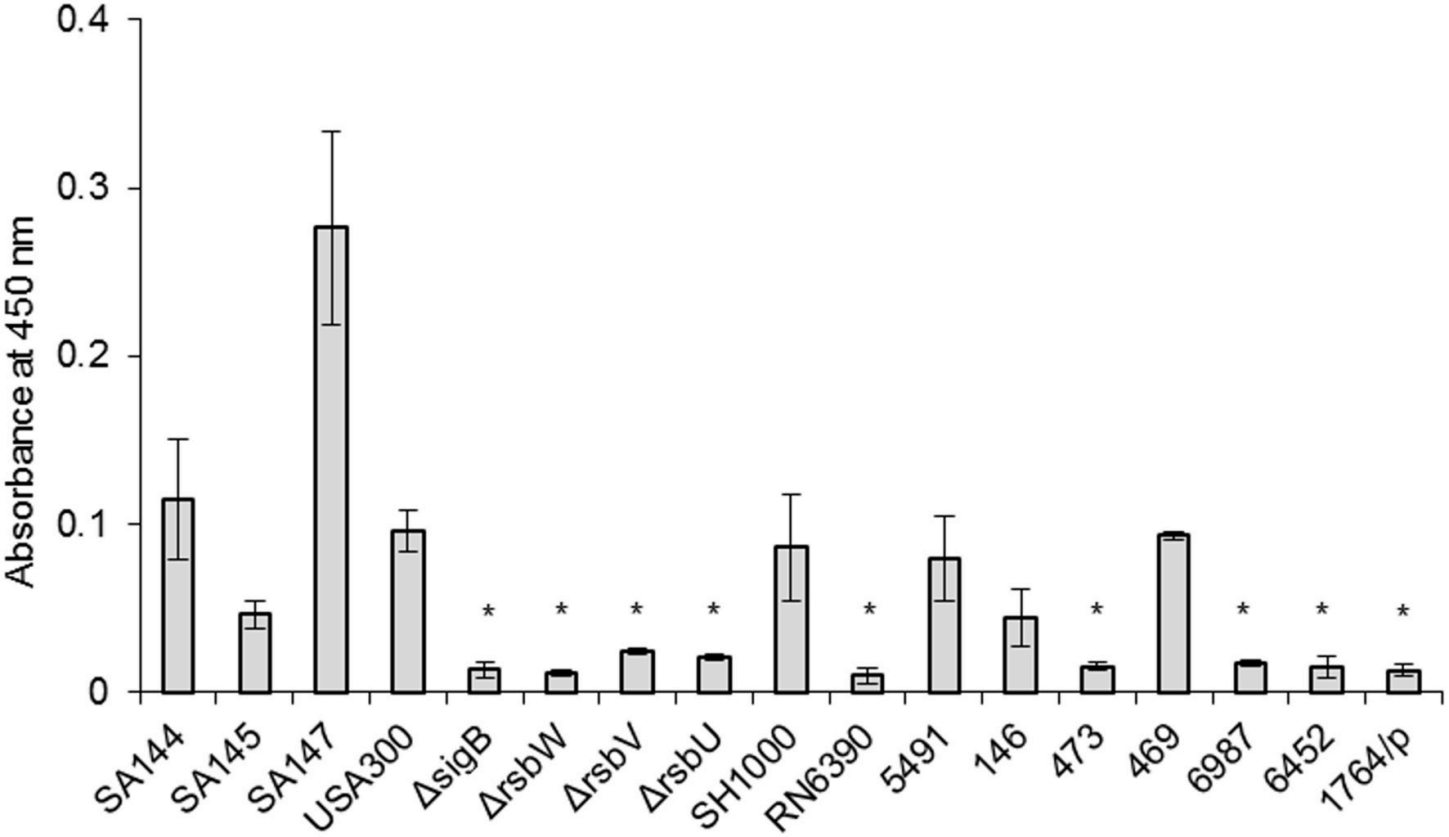
**Carotenoid levels**. The results shown are the average of three independent experiments, and error bars represent standard deviation. Asterisks above white bars indicate significant differences relative to wild type *S. aureus* strain SA144 after 24 h (*p* < 0.05).

Methanol extracts from *S. aureus* contain a mixture of various pigments with absorbancemaximum around λ = 450 nm, ranging from a pale yellow 4.4′-diapo-ζ-carotene to a red 4.4′-diaponeurosporenal. These constitute the intermediate products of the staphyloxanthin (STX) synthesis pathway and are generally referred to as carotenoids (Marshall and Wilmoth, 1981). RsbU-activated σ^B^ drives the expression of membrane-localized STX in *S. aureus* (Giachino et al., 2001; Olivier et al., 2009). Therefore, we used STX levels as another functional test of σ^B^ activity. As a reference system, we used a subset of isogenic strains differing in pigmentation status (SA144, SA145, SA147; see Table 1). We measured carotenoid levels in all analyzed strains (Figure 4). Reference strains with an intact *sigB* operon, namely USA300 and SH1000, accumulated similar amounts of carotenoids as strain SA144. As expected, RN6390 with non-functional RsbU accumulated significantly less carotenoids than SA144. Similarly, each Δ*rsbUVWsigB* isogenic mutant accumulated significantly less carotenoids compared to reference SA144. The level of carotenoids in clinical isolates stayed in compliance with *asp23* transcript-coupled σ^B^ activity. As expected, carotenoid synthesis was strongly inhibited in isolates 473, 6987, 6452, and 1764/p, where decreased activity of σ^B^ was observed (Figure 3). Extracts of strains 5491, 146, and 469 revealed similar absorbance values as the reference wild type SA144 strain, indicating relatively high carotenoid levels.

### Staphyloxanthin production is important but is not the only mechanism of bacterial cells response to PACT

Carotenoid pigments are broad-range reactive oxygen species scavengers, showing the ability to neutralize free radicals as well as efficiently quench singlet oxygen (^1^O_2_) (Clauditz et al., 2006). To assess if the lack of pigmentation is crucial and sufficient for determining PACT outcome, we first assessed the phototoxic effect of PPArg_2_, ZnPC and RB on reference strains SA144, SA145, and SA147, in which carotenoid production is regulated at the level of the *crtOPQMN* operon (Table 5). Notably, in all three cases of the studied PSs, the non-pigmented strain SA145 was the most vulnerable to PACT treatment compared to its pigmented counterparts (SA144, SA147). A significant bactericidal effect or total eradication was observed after RB and ZnPC treatments but not PPArg_2_. Additionally, the ZnPC bactericidal effect on wild type strain SA144 was abolished in the carotenoid-overproducing strain SA147. A similar trend could be seen in the case of RB; however, the values of survival reduction for this PS were lower. Based on the obtained results, pigmentation levels correlated with photokilling efficacy with the three PSs used in our experimental conditions. In the case of clinical isolates, carotenoid levels correlated with RsbU-dependent σ^B^ activity and may have a vital contribution to PACT outcome. Nevertheless, pigment levels remained relatively high in the PACT-sensitive strains 146 and 469, with undisturbed σ^B^ activity, suggesting that other mechanisms must be involved.

**Table 5:** **Phototoxic effect on ***S. aureus*** reference strains differing in pigmentation status**.

**Strain**	**Mean reduction of survival**^a^ **(log**_10_ **CFU) ± SD**		
	**PPArg_2_^b^**	**ZnPC^b^**	**RB^c^**
SA144 (wild type pigmentation)	0.58 ± 0.28	**3.67 ± 0.84**	1.22 ± 0.11
SA145 (lack of pigmentation)	2.81 ± 0.52	**5.97 ± 0.26**	**3.94 ± 0.90**
SA147 (high pigmentation)	0.47 ± 0.10	0.34 ± 0.30	0.37 ± 0.17

a *The values were calculated by subtracting log_10_ CFU/ml of treated samples from those of untreated controls (0 J/cm^2^; 0 μM PS). At least three biological replicates were used for calculation of the mean reduction values. SD–Standard Deviation. Underlined values indicate statically significant reduction relative to light control (20 J/cm^2^; 0 μM PS) (p < 0.05). Bold values indicate a bactericidal effect (>3 log_10_ reduction units)*.

b *Photosensitizer excited with the red light (maximal intensity at λ_max_ 627 nm). Concentrations used: PPArg_2_ 20 μM; ZnPC 5 nM*.

c *0.1 μM RB excited with the green light (maximal intensity at λ_max_ 520 nm)*.

### Membrane fluidity reflects vulnerability to PACT

**Figure 5:**
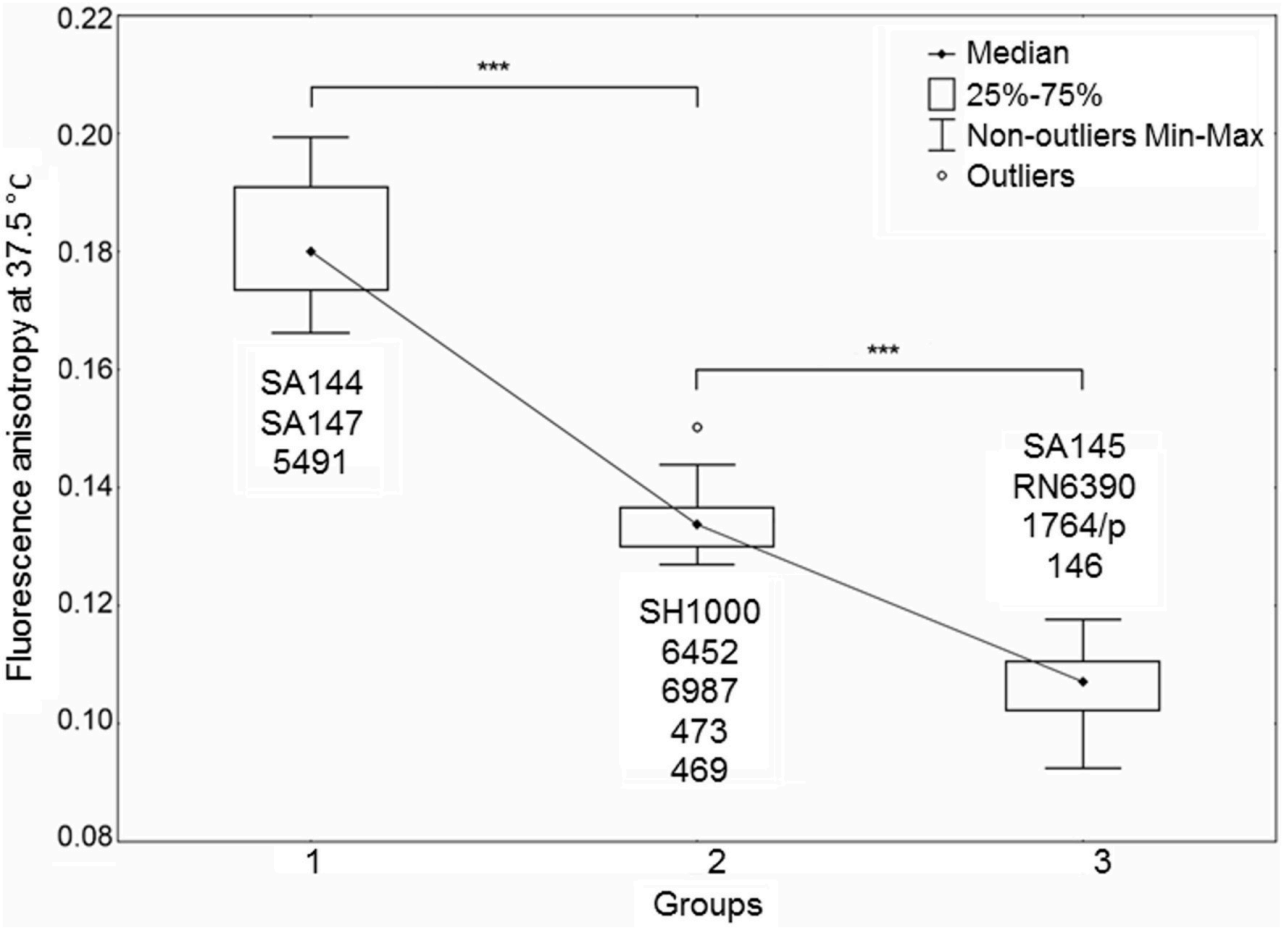
*****Staphylococcus aureus*** cell membrane fluidity**. The plot presents three distinct groups of strains differing in membrane fluidity. The datasets of three independent experiments for each strain are presented. Significant differences between groups at a level of *p* < 0.001 are marked with asterisks. An inverse relationship occurred between the fluorescence anisotropy and cell membrane fluidity. Outlier—a single measurement for strain 6452.

Bacterial membrane fluidity, among other factors, such as protein content, also reflects changes in staphyloxanthin level (Mishra et al., 2011). Apart from providing antioxidant defense, the STX biosynthesis pathway shares similarities with that of human cholesterol, suggesting similar roles in maintaining cell membrane fluidity. We measured temperature-dependent fluorescence anisotropy (*r*) in DPH-labeled bacterial cell membranes. An inverse relationship occurs between measured DPH fluorescence anisotropy and membrane fluidity (Mishra et al., 2011). Statistical analysis of *r*-values revealed a strong dependence of all measurements within the studied temperature range, presenting the same trend in anisotropy drop upon heating (Supplementary Figure 3). Figure 5 presents the *r*-values for optimal growth temperature of *S. aureus* at 37.5°C. The statistical analysis resulted in distinguishing three groups of strains with distinct fluorescence anisotropy values (Supplementary Figure 3). Analysis of the strains SA144, SA145 and SA147 confirmed a pronounced difference between pigmented and non-pigmented cells. High *r*-values (low membrane fluidity) were observed for pigmented strains SA144 and SA147 (Figure 5, Group 1). The reference pair of SH1000 and RN6390 also revealed significantly different *r*-values; however, despite the similar carotenoid content in SH1000 and the strains ranked in the first group (SA144, SA147, 5491), the former was included in Group 2. This leads to the assumption that STX is not the only factor affecting overall membrane fluidity. The third group (Figure 5, Group 3) presenting the highest membrane fluidity included the strains SA145, RN6390 and 1764/p, which are characterized by poor pigmentation, and strain 146. In the case of strains 146 (Group 3) and 469 (Group 2), which presented neither the abolished σ^B^ activity nor significantly inhibited carotenoid accumulation, we suppose that a high membrane fluidity of unknown background is the factor sensitizing those isolates to photosensitizer action.

## Discussion

The general mechanism of PACT action is multifactorial in that it can act on various targets in the cell (proteins, lipids, DNA). The properties of a photosensitizer dictate how efficiently it will act on a particular species of bacteria. On the other hand, even highly efficient PSs will act on different strains of the same species with varied efficacy. Notably, PACT was shown to be effective not only in inactivation of *S. aureus* but also in the degradation of virulence factors produced by this microorganism (Bartolomeu et al., 2016). We were interested in exploring if σ^B^, or other elements functionally connected with σ^B^ (STX, carotenoids, membrane fluidity), may play any role in the process of PACT. Out of six different PSs used in *S. aureus* photoinactivation experiments, three discriminated the wild type strain USA300 from the *sigB* operon mutants upon illumination (Table 2). This observation indicates that σ^B^ operon function is important for PACT outcome. Notably, we identified the *rsbU* gene as the most important element in the *sigB* operon as the analyzed PACT-sensitive strains had mutations within the *rsbU* sequence.

Not all photosensitizers included in the analysis showed diverse patterns of action with respect to a genetic background of *S. aureus* strains. Toluidine blue O and TMPyP were equally ineffective toward both wild type and σ^B^-impaired mutants at herein applied conditions. A weak phototoxic effect, however, cannot be linked to chosen concentrations of PSs. According to the literature, 25 μM TBO is the most effective toward *S. aureus* and yet is below the EC_50_ value for human keratinocytes (Kossakowska et al., 2013). For tetra-cationic porphyrins concentrations as low as 1 or 5 μM are effective toward vulnerable *S. aureus* strains (Ke et al., 2014; Pereira et al., 2014), and 25 μM was shown to be as effective as higher concentrations (Hanakova et al., 2014). With regards to the mechanism of action, both PSs are potent ^1^O_2_ producers (Bacellar et al., 2014; Taraszkiewicz et al., 2015), therefore representing predominant type II photoreactions. Literature data showing interactions of PS with *S. aureus* describe TBO as a substrate for NorA efflux pump, preventing efficient intracellular PS accumulation (Tegos et al., 2008). Its photodynamic activity in the extracellular environment has been confirmed by a significant drop in phototoxic effect upon washing out extra PS (Demidova and Hamblin, 2005). As a biological effect, TBO induces a break in contact between the cell wall and the membrane of *S. aureus* (Sahu et al., 2009). A second compound, TMPyP, belongs to cationic photosensitizers, which interact with negatively-charged bacterial cell surface via self-promoted accumulation (Lambrechts et al., 2004). However, our observations presented elsewhere (Kossakowska-Zwierucho et al., 2015) indicate that TMPyP does not accumulate in *S. aureus* cells, as measured spectrophotometrically for strains SH1000 and RN6390. The background for this observation is unknown and may result either from a large molecular structure of TMPyP preventing effective uptake, which was also discussed elsewhere in a context of hindered biofilm penetration (Cieplik et al., 2013), or the presence of a yet unidentified efflux system. However, such research has not been published thus far. Nevertheless, similar to TBO, TMPyP seems to act in the extracellular environment rather than within the cell. A fullerene derivative, although more potent than TBO and TMPyP at herein applied conditions, did not show discriminative activity toward a wild type or mutant reference strain. Fullerenes, as opposed to other PSs used in the study, represent predominantly type I photoreactions, acting mainly via the generation of superoxide anion (Mizuno et al., 2011), which was also confirmed for the fulleropyrrolidine derivative used in our experiments (Grinholc et al., 2015). Equal efficacy toward analyzed strains may suggest that the σ^B^-mediated response to photodynamic stress is not crucial in defense against this particular oxidative factor. On the other hand, a set of compounds, including protoporphyrin diarginate, zinc phthalocyanine and rose bengal, showed a high efficacy toward σ^B^-impaired and non-pigmented *S. aureus* strains. PPArg_2_, a water soluble derivative of protoporphyrin IX, is a potent producer of ^1^O_2_ (Fernandez et al., 1997; Ye et al., 2003). It accumulates in *S. aureus* cells and can easily be measured at a chosen concentration. This process has been characterized to occur either by passive penetration or (to a greater extent) by active transport accompanied by as yet unknown proteins (Grinholc et al., 2010). ZnPC, also acting predominantly via a type II photodynamic mechanism (Ogunsipe et al., 2003), is a potent, albeit strongly hydrophobic, compound, which brings difficulties in its solubility. In our experimental conditions, this disadvantage was inconclusive because concentrations as low as 5 nM were efficient for photokilling of vulnerable strains. Such low concentrations prevent credible measurements of this compound accumulation in bacterial cells using the herein described methods. However, ZnPC has been characterized elsewhere as active against *S. aureus* even after washing out the unbound PS (Ke et al., 2014). This suggests that an intracellular accumulation of ZnPC occurs. Another compound active against σ^B^-defective *S. aureus* mutants is RB. It is an anionic fluorescein derivative of very high singlet oxygen yield (Wilkinson et al., 1993). With regards to intracellular accumulation, a 0.1 μM concentration used in this study also hindered the uptake measurement by spectrophotometry. However, according to the literature, its photodynamic activity against *S. aureus* is similar before and after washing out unbound PS (Demidova and Hamblin, 2005). This indicates that, despite a negative charge, RB can effectively accumulate in *S. aureus*, which may be linked to its lipophilic nature (Dahl et al., 1989; Cieplik et al., 2014). Based on the above PSs' characteristics, a trend can be observed, in which σ^B^–defective *S. aureus* strains are easily killed in the presence of cell-bound (or intracellularly accumulated) photosensitizers of high ^1^O_2_ yield. This observation was confirmed using another pair of reference strains (SH1000 and RN6390) presenting different genetic backgrounds for RsbU dysfunction (Table 2).

We were interested in *sigB* analysis, as this is a crucial element of bacterial defense against various stress conditions. Photoinactivation generates oxidative stress in bacterial cells; thus, it was of interest to investigate the relationship between PACT outcome and *sigB* activity. First, we showed that inactivation of any genes in the *sigB* operon was important in sensitizing *S. aureus* to PACT (Table 2). Notably, we confirmed this observation based on screening for aberrations in the *sigB* operon of clinical *S. aureus* isolates described elsewhere (Grinholc et al., 2008) as sensitive to PACT. Six isolates were confirmed to be effectively killed by at least two out of three applied photosensitizers (Table 3). One clinical isolate (5491) insensitive to the herein applied photodynamic conditions was also included in the analyses. Interestingly, we identified multiple aberrations accumulated within the *rsbU* gene of PACT-vulnerable strains by DNA sequencing (Table 4). However, the lack of a structural model of the *S. aureus* RsbU protein hindered the picture of identified mutations. Therefore, bioinformatic analyses allowed us to predict the 3D structure of single-chain RsbU of *S. aureus*. Mutations indicated in Figure 1 and Table 4 are localized to all three domains of RsbU and include single amino acid substitutions, probable protein truncations due to an early stop codon and insertion of IS256—a mobile element occurring in multiresistant staphylococci and enterococci (Kozitskaya et al., 2004). Analysis of *asp23* transcript levels, a direct marker of σ^B^ activity, allowed us to assess whether identified mutations impair RsbU and therefore σ^B^ function. In four out of six mutants, *asp23* transcript levels were decreased. This observation is consistent with severe aberrations, including an IS256 insertion in position 321, and a probable RsbU truncation, and indicate that substitutions A230T and A276D diminish RsbU and therefore σ^B^ activity. Both single substitutions affected alanine replaced by a larger polar threonine and negatively charged aspartic acid. Those changes localized to positions of hypothetical significance for protein structure and activity, specifically in a turn between β-sheets 7 and 8 of the phosphatase domain (A230) and in close proximity to an aspartate residue D271 (A276) participating in metal ion cofactor binding. Verification of this hypothesis would require additional experiments, i.e., complementation of mutants with a functional copy of *rsbU* and/or site-directed mutagenesis of a functional RsbU. In general, the described mutations localized to the C-terminal phosphatase domain, indicating their crucial role in σ^B^ activation. A probable RsbU truncation and a lack of C-terminal domain also diminished protein activity. The actual role of the RsbU N-terminal domain remains elusive because no upstream regulatory mechanism has thus far been described in *S. aureus*. These observations need to be further verified.

Aside from *asp23* transcript levels, a clear correlation exists between σ^B^ activity and staphylococcal carotenoid levels (Figure 4). In all strains with impaired σ^B^ function, we observed strong inhibition of carotenoid production, which was undisturbed when σ^B^ was active. Because STX production is dependent on σ^B^ activity, we wanted to check if pigment level was a sufficient factor determining *S. aureus* sensitivity to PACT. Photoinactivation experiments revealed a correlation between pigment levels and PACT efficacy, where non-pigmented strains were the most vulnerable to treatment with all three PSs, whereas pigment-overproducing strains were completely insensitive to the applied treatment (Table 5). The relatively low sensitivity of non-pigmented strains to PPArg_2_ could result from limited PS accumulation in this set of strains, as was confirmed spectrophotometrically (Supplementary Figure 2). Similar decreases in PS accumulation could also explain why clinical isolate 6452, despite having an obvious RsbU dysfunction and low carotenoid levels, was not effectively killed in the presence of PPArg_2_ (Table 3). The obtained results indicate that STX production is a significant element of σ^B^-dependent mechanism in response to PACT.

However, our observation did not explain the high susceptibility of the two clinical isolates presenting undisturbed σ^B^ activity and carotenoid production (469 and 146) to PACT. A weak response of strain 469 to RB may be linked to its relatively high carotenoid content, confirming a vitality of antioxidant pigments in RB-based treatment. In this context, however, it does not explain the strain's vulnerability to PPArg_2_ and ZnPC. A putative sensitizing factor could be their high cell membrane fluidity. The conducted experiments showed a correlation between cell membrane fluidity and STX content of the SA144 reference variants. However, strains 469 and 146 located in Groups 2 and 3 did not fit this picture. The unusual extent of cell membrane fluidity was reached in strain 146, which was highly sensitive to all applied PSs. The obtained results suggest that bacterial cell membrane fluidity, resulting from various factors aside from staphyloxanthin and membrane-associated Asp23 content (Müller et al., 2014), may be a significant element sensitizing *S. aureus* to PACT.

## Conclusion

In summary, we describe RsbU-dependent σ^B^ activity as a significant element of the *S. aureus* response to photodynamic therapy with the use of intracellularly accumulating, highly efficient ^1^O_2_-producing photosensitizers. For the first time, we present the structure of the *S. aureus* RsbU protein monomer based on computational predictions. The results of molecular modeling and applied functional tests suggest RsbU as an important factor for σ^B^ activation and PACT response, however, further experiments are needed to clearly confirm it. We propose that carotenoid levels are a reliable biochemical marker of σ^B^ activity and emphasize the role of staphyloxanthin in the *S. aureus* response to PACT. We propose enhanced bacterial cell membrane fluidity as a hallmark of *S. aureus* susceptibility to PACT, however this is not the only factor contributing to PACT-vulnerable phenotype.

## Author contributions

MK performed the experiments, analyzed the results, drafted the manuscript. RK prepared molecular model of RsbU protein and drafted the manuscript. KB edited and drafted the manuscript. JN conceived the study, carried out the experimental work, analyzed the results and drafted the manuscript. All authors read and approved the final manuscript.

## Funding

The research was funded by the National Science Centre grant no. 1640/B/P01/2010/39 (JN) and University of Gdansk grant for young investigators no. 538-M036-B139-13 (MK).

### Conflict of interest statement

The authors declare that the research was conducted in the absence of any commercial or financial relationships that could be construed as a potential conflict of interest. The reviewer EF and handling Editor declared their shared affiliation, and the handling Editor states that the process nevertheless met the standards of a fair and objective review.
