# Supplementary material for: Factors Determining Staphylococcus aureus Susceptibility to Photoantimicrobial Chemotherapy: RsbU Activity, Staphyloxanthin Level, and Membrane Fluidity
Source: Front Microbiol. 2016 Jul 19;7:1141. doi: 10.3389/fmicb.2016.01141 (PMC4949386; doi:10.3389/fmicb.2016.01141)
Supplement: Supplementary file 1 [file Image1.PDF]

Supplementary Figure 1

Factors determining *Staphylococcus aureus* susceptibility to photoantimicrobial chemotherapy: RsbU activity, staphyloxanthin level and membrane fluidity.

Monika Kossakowska-Zwierucho, Rajmund Kaźmierkiewicz, Krzysztof P. Bielawski, Joanna Nakonieczna*

*** Correspondence:** joanna.nakonieczna@biotech.ug.edu.pl

Phone: 0048 58 5236332

Fax: 0048 58 5236426

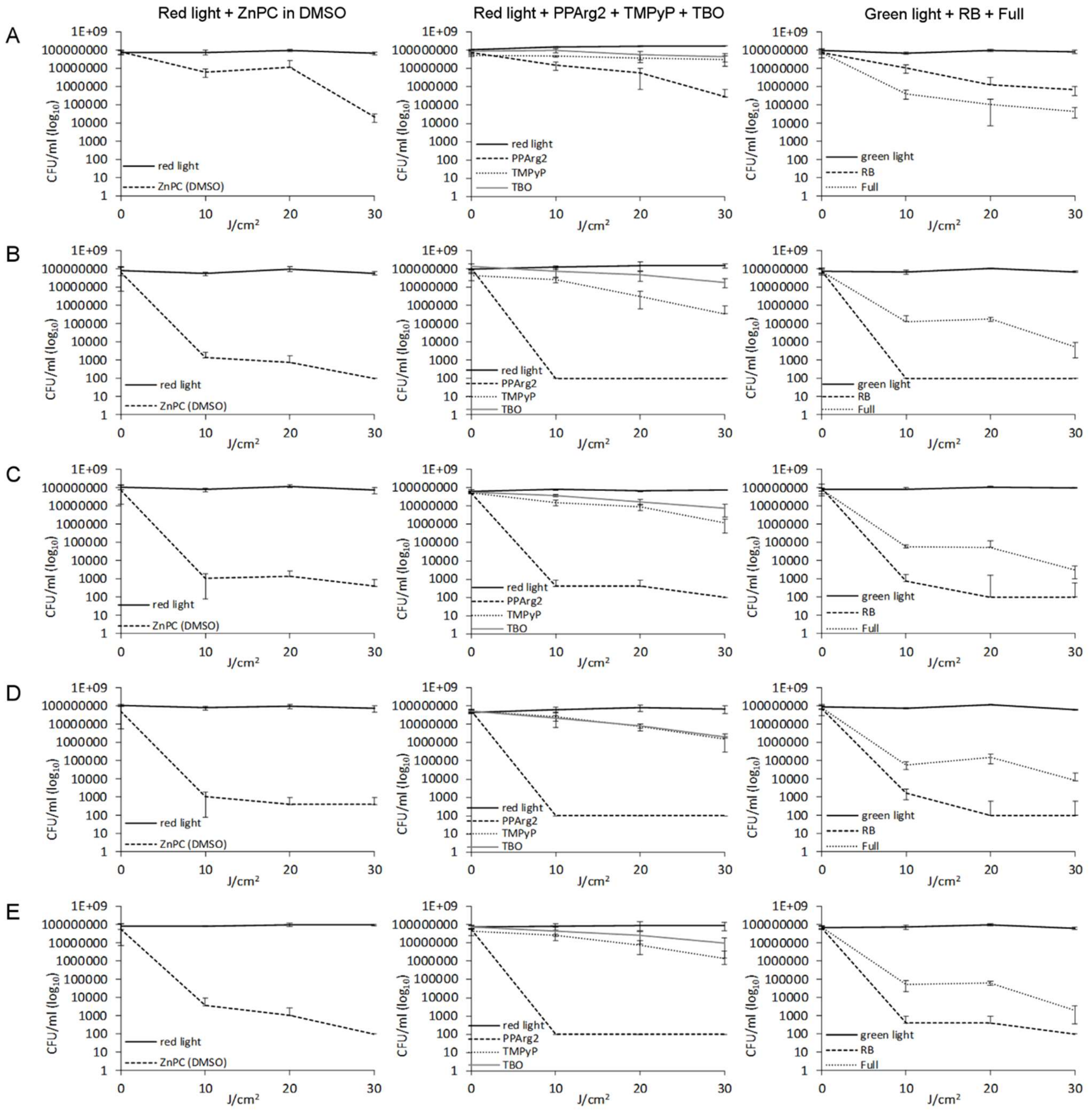

Supplementary Figure 1. Survival curves of photodynamically treated reference *S. aureus* strains. (**A**) USA300 wild type strain, (**B**) *ΔrsbU*, (**C**) *ΔrsbV*, (**D**) *ΔrsbW*, (**E**) *ΔsigB*. Columns show survival upon treatment with: red light and phthalocyanine (ZnPC in 0.5% final concentration of DMSO); red light and protoporphyrin IX diarginate ($PPArg_2$), 5.10.15.20-tetrakis(1-methyl-4-pyridinio)porphyrin tetra(*p*-toluenesulfonate) (TMPyP) and toulidine blue O (TBO); green light and rose bengal (RB) and fulleropyrrolidine (Full in 0.1% final concentration of DMSO). Red light corresponds to a wavelength of λ_{max} 627 nm, green light corresponds to λ_{max} 520 nm. The following concentrations were used: ZnPC at 5 nM; PPArg2, TMPyP and TBO at 20 µM; RB at 0.1 µM and Full

at 1 μM. Each experiment was done at least in triplicate, error bars show standard deviations (SD). Red light and green light controls indicate cells incubated in a solution of 0.5% (in the case of column "Red light + ZnPC in DMSO") or 0.1% (in the case of column "Green light + RB + Full) of DMSO.
