# Supplementary material for: Factors Determining Staphylococcus aureus Susceptibility to Photoantimicrobial Chemotherapy: RsbU Activity, Staphyloxanthin Level, and Membrane Fluidity
Source: Front Microbiol. 2016 Jul 19;7:1141. doi: 10.3389/fmicb.2016.01141 (PMC4949386; doi:10.3389/fmicb.2016.01141)
Supplement: Supplementary file 2 [file Image2.PDF]

Supplementary Figure 2

Factors determining *Staphylococcus aureus* susceptibility to photoantimicrobial chemotherapy: RsbU activity, staphyloxanthin level and membrane fluidity.

Monika Kossakowska-Zwierucho, Rajmund Kaźmierkiewicz, Krzysztof P. Bielawski, Joanna Nakonieczna*

*** Correspondence:** joanna.nakonieczna@biotech.ug.edu.pl

Phone: 0048 58 5236332

Fax: 0048 58 5236426

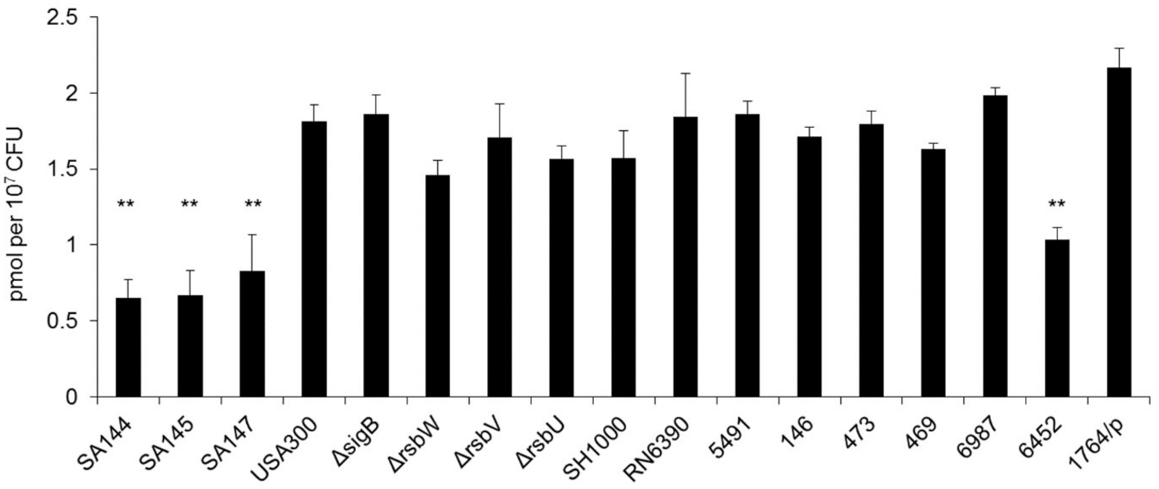

Supplementary Figure 2. Protoporphyrin IX diarginate uptake.

Bacterial cells were incubated with 20 µM $PPArg_2$ in the dark at 37 °C for 30 min and treated as described in Materials and Methods. Cells were then centrifuged and $PPArg_2$ concentrations in the supernatant were measured. The obtained values were subtracted from the initial concentration of 3.2 nmol per 10^7 cells. The results shown are the average of at least three independent experiments, and error bars represent standard deviation. Significance at a level of $p<0.05$ is marked with asterisks. The names of particular strains are indicated on the graph.
