# Supplementary material for: Factors Determining Staphylococcus aureus Susceptibility to Photoantimicrobial Chemotherapy: RsbU Activity, Staphyloxanthin Level, and Membrane Fluidity
Source: Front Microbiol. 2016 Jul 19;7:1141. doi: 10.3389/fmicb.2016.01141 (PMC4949386; doi:10.3389/fmicb.2016.01141)
Supplement: Supplementary file 3 [file Image3.PDF]

Supplementary Figure 3

Factors determining *Staphylococcus aureus* susceptibility to photoantimicrobial chemotherapy: RsbU activity, staphyloxanthin level and membrane fluidity.

Monika Kossakowska-Zwierucho, Rajmund Kaźmierkiewicz, Krzysztof P. Bielawski, Joanna Nakonieczna*

*** Correspondence:** joanna.nakonieczna@biotech.ug.edu.pl

Phone: 0048 58 5236332

Fax: 0048 58 5236426

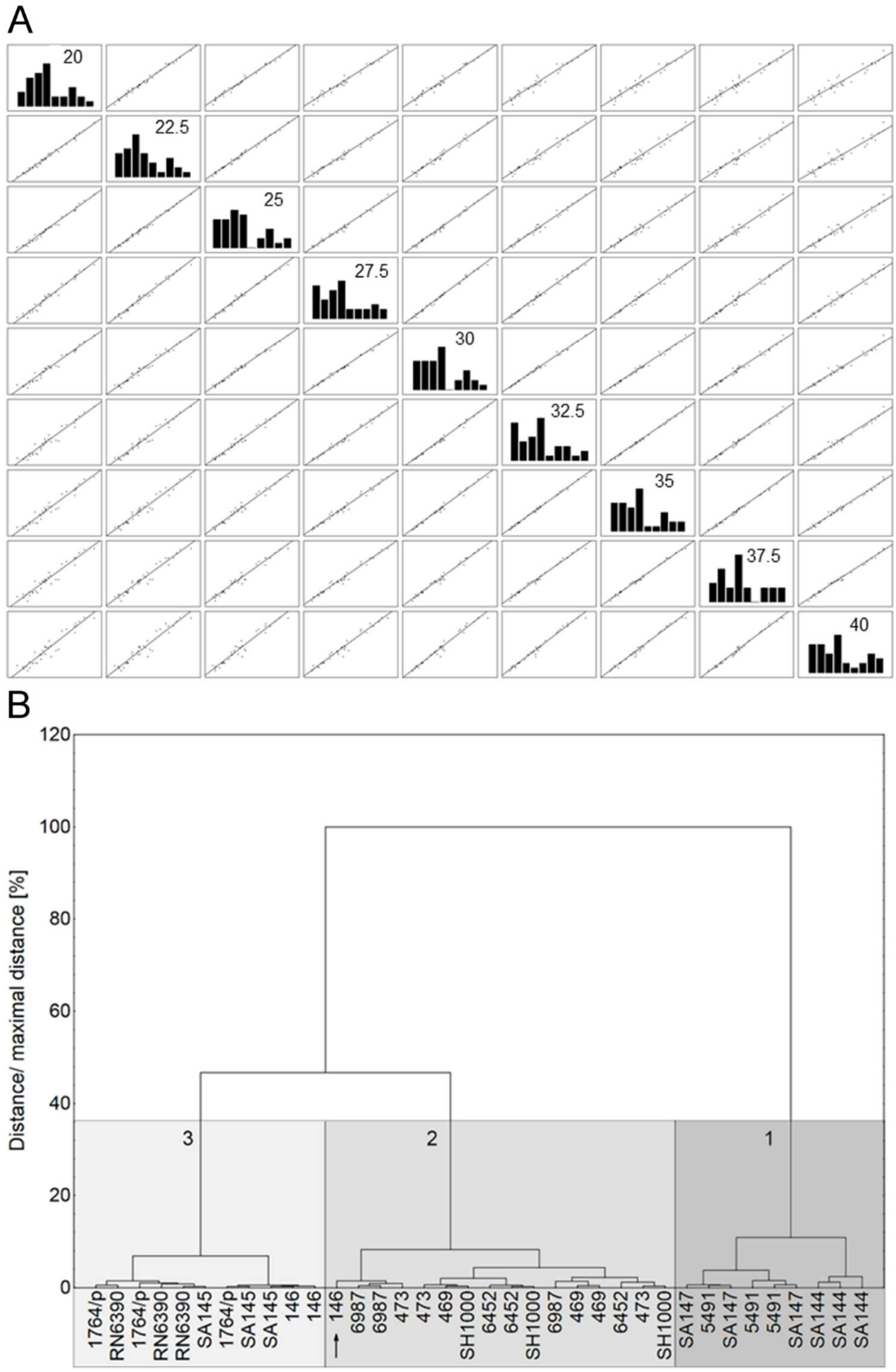

Supplementary Figure 3. Cluster tree of fluorescence anisotropy data.

The values of anisotropy (r) were measured three times for each *Staphylococcus aureus* strain (indicated below the graph). Grouping of fluorescence anisotropy data included analysis of correlation matrix within a whole temperature range followed by hierarchic clustering based on Ward

agglomeration and Manhattan distance calculation methods using STATISTICA 10 software (StatSoft Inc. 2011, USA). (A) Graphical representation of correlation matrix. Analysis of data structure including nine measurement points for each strain replicate revealed a high correlation throughout the whole temperature range (20 to 40°C). (B) The applied data grouping resulted in emergence of three sets of strains statistically differing within a whole temperature range, where Group 1 presents the highest *r* values and Group 3 the lowest. Strain 146 (indicated by an arrow), allocated in two groups (two replicates in Group 3 and one replicate in Group 2), was assumed for further analyses to be contained in Group 3.
